# 5-ALA fluorescence of cerebral metastases and its impact for the local-in-brain progression

**DOI:** 10.18632/oncotarget.11488

**Published:** 2016-08-22

**Authors:** Marcel A. Kamp, Igor Fischer, Julia Bühner, Bernd Turowski, Jan Frederick Cornelius, Hans-Jakob Steiger, Marion Rapp, Philipp J. Slotty, Michael Sabel

**Affiliations:** ^1^ Department of Neurosurgery, Medical Faculty, Heinrich-Heine-University, Düsseldorf, D-40225 Düsseldorf, Germany; ^2^ Division of Informatics and Statistics, Department of Neurosurgery, Medical Faculty, Heinrich-Heine-University, Düsseldorf, D-40225 Düsseldorf, Germany; ^3^ Institute for Diagnostic and Interventional Radiology, Medical Faculty, Heinrich-Heine-University, Düsseldorf, D-40225 Düsseldorf, Germany

**Keywords:** 5-aminolevulinic acid, cerebral metastases, recurrence, in-brain-progression, overall survival

## Abstract

Aim of the present study was to analyze the oncological impact of 5-ALA fluorescence of cerebral metastases. A retrospective analysis was performed for 84 patients who underwent 5-ALA fluorescence-guided surgery of a cerebral metastasis. Dichotomized fluorescence behavior was correlated to the histopathological subtype and primary site of the metastases, the degree of surgical resection on an early postoperative MRI within 72 hours after surgery, the local in-brain-progression rate and the overall survival. 34/84 metastases (40.5%) showed either strong or faint and 50 metastases (59.5%) no 5-ALA derived fluorescence. Neither the primary site of the cerebral metastases nor their subtype correlated with fluorescence behavior. The dichotomized 5-ALA fluorescence (yes vs. no) had no statistical influence on the degree of surgical resection. Local in-brain progression within or at the border of the resection cavity was observed in 26 patients (30.9%). A significant correlation between 5-ALA fluorescence and local in-brain-progression rate was observed and patients with 5-ALA-negative metastases had a significant higher risk of local recurrence compared to patients with 5-ALA positive metastases. After exclusion of the 20 patients without any form of adjuvant radiation therapy, there was a trend towards a relation of the 5-ALA behavior on the local recurrence rate and the time to local recurrence, although results did not reach significance anymore. Absence of 5-ALA-induced fluorescence may be a risk factor for local in-brain-progression but did not influence the mean overall survival. Therefore, the dichotomized 5-ALA fluorescence pattern might be an indicator for a more aggressive tumor.

## INTRODUCTION

Cerebral metastasis are the most common cerebral neoplasms and, for different reasons, the incidence is increasing [1]. The surgical resection of single cerebral metastases is a key element in a multimodal therapeutic concept (level I evidence) [2–5]. Surgical standard is a microsurgical, white-light, microscope assisted circumferential stripping of the tumor from the adjacent brain tissue [6]. The major goal is to achieve local tumor control by a complete sugical resection with low morbidity and mortality. However, surgery alone is not sufficient to achieve local control in about 50% of patients [7–9]. An infiltrative growth pattern of cerebral metastases and small, unintended residual tumor parts even after intended gross-total tumor resection have been discussed as reasons for the high local recurrence rate [10–15].

Fluorescence-guided resection of cerebral metastases has been discussed as an approach to visualize residual tumor tissue and maximize the extent of the surgical resection. The technique of 5-aminolevulinic acid (5-ALA) guided resections was first introduced in malignant glioma. 5-ALA is accumulated and converted to the fluorescent agent protoporphyrin IX (PpIX) in malignant gliomas and allows a selective visualization of tumor tissue after blue-light illumination. This technique is used for its highly sensitive and highly specific intraoperative tumor detection and can increase the percentage of complete resections and the progression free survival when compared to conventional white-light surgery [16–18]. Some but not all cerebral metastases exhibit a similar 5-ALA-derived fluorescence as seen in malignant glioma [15, 19, 20]. In the two largest series analyzing this phenomenon, 47.7% and 38.5% resp. of cerebral metastases showed no intraoperative ALA-induced fluorescence [15, 20].

Besides general feasibility, the oncological impact of 5-ALA use in cerebral metastases surgery remains unclear so far. Aim of the present study was (1) to correlate 5-ALA-derived fluorescence of cerebral metastases with the extent of surgical resection, (2) the primary site and histopathological subtype, (3) the local-recurrence rate and (4) the overall survival.

## RESULTS

### Patients

In total, 84 patients suffering from cerebral metastatic spread were identified who underwent intraoperative 5-ALA estimation during microsurgical resection of a cerebral metastasis. 46 patients were female, 38 male (female to male ratio; 1.2:1). Mean age was 61.8 years (range: 32–83 years). 62 patients suffered from an adenocarcinoma, 7 from small cell cancer, 5 from clear cell cancer, 6 from squamous cell cancer and 4 from malignant melanoma. Non-small cell lung cancer (NSCLC) could be identified as primary tumor in 44 patients, breast cancer in 11 patients, small-cell lung cancer (SCLC) or renal cancer in 6 patients each, carcinoma of unknown primary in 5 patients, malignant melanoma, carcinoma arising from the gastro-intestinal tract or the uro-genital tract in four patients each. Clinical data are summarized in Table 1.

**Table 1:** Summary of clinical data

		*n*	%
	number of patients	84	
	number of metastases	84	
**Age**			
	median	63 y	
	mean	61.8 y	
	range	32–83 y	
**Gender**			
	female	46	54.8%
	male	38	45.2%
**Histology**			
	adeno-CA	62	73.8%
	small cell CA	7	8.3%
	clear cell CA	5	6.0%
	squamous cell CA	6	7.1%
	malignant melanoma	4	4.8%
**Primary cancer**			
	NSCLC	44	52.3%
	SCLC	6	7.1%
	malignant melanoma	4	4.8%
	breast cancer	11	13.1%
	gastro-intenstinal cancer	4	4.8%
	urogenital cancer	4	4.8%
	renal cancer	6	7.1%
	carcinoma of unknown primary	5	6.0%
**Adjuvant radiation therapy**			
	whole-brain-radiation theray	54	64.3%
	local	10	11.9%
	none	20	23.8%
**5-ALA derived fluorescence**			
	yes	34	40.5%
	no	50	59.5%
**Degree of surgical resection**			
	complete	47	56%
	incomplete	15	17,8%
	questionable	22	26,2%
**Incidence of local in-brain-progression (pts.)**			
	total	26	30.9%
	ALA-positive	6	7.1%
	ALA-negative	20	23.8%

The vast majority of patients (52 pts; 61.9%) received an adjuvant whole-brain radiation therapy (WBRT)–combined with stereotactic radiosurgery on an arguable tumor rest in two additional patients. Ten patients underwent stereotactic radiosurgery of the resection cavity with a safety margin of additional 5 mm. 20 patients (23.8%) received no form of additional radiotherapy.

No permanent side effects of 5-ALA were observed. 5 patients suffered from a transient erythema after unintentional exposure to daylight, which resolved within 3 days. Liver enzymes, serum leucocytes were not significantly change at the third postoperative day as compared to the preoperative values (aspartate transaminase: preoperative: 36.1 ± 3.1 U/l, postoperative: 37 ± 3.2 U/l, *p* = 0.91, Welch Two Sample *t*-test; 95– CI: −4.7–5.2; alanine transaminase: preoperative: 23.2 ± 1.7 U/l, postoperative: 26.9 ± 1.8 U/l, *p* = 0.84, Welch Two Sample *t*-test; 95-CI: −9.5–7.8; serum leucocytes: preoperative: 11.8 ± 5.1 × 1000/μl, postoperative: 11.5 ± 4.3 1 × 1000/μl, *p* = 0.75, Welch Two Sample *t*-test; 95-CI: −1.2–1.7). Postoperatively, serum hemoglobin levels were postoperatively significantly lower than before surgery (preoperative: 13.6 ± 0.17 g/dl, postoperative: 12.6 ± 0.2 g/ dl; *p* < 0.0001; Fisher's Exact Test; 95-CI: 0.67–1.32).

### 5-ALA derived fluorescence and extend of surgical resection

**Figure 1:**
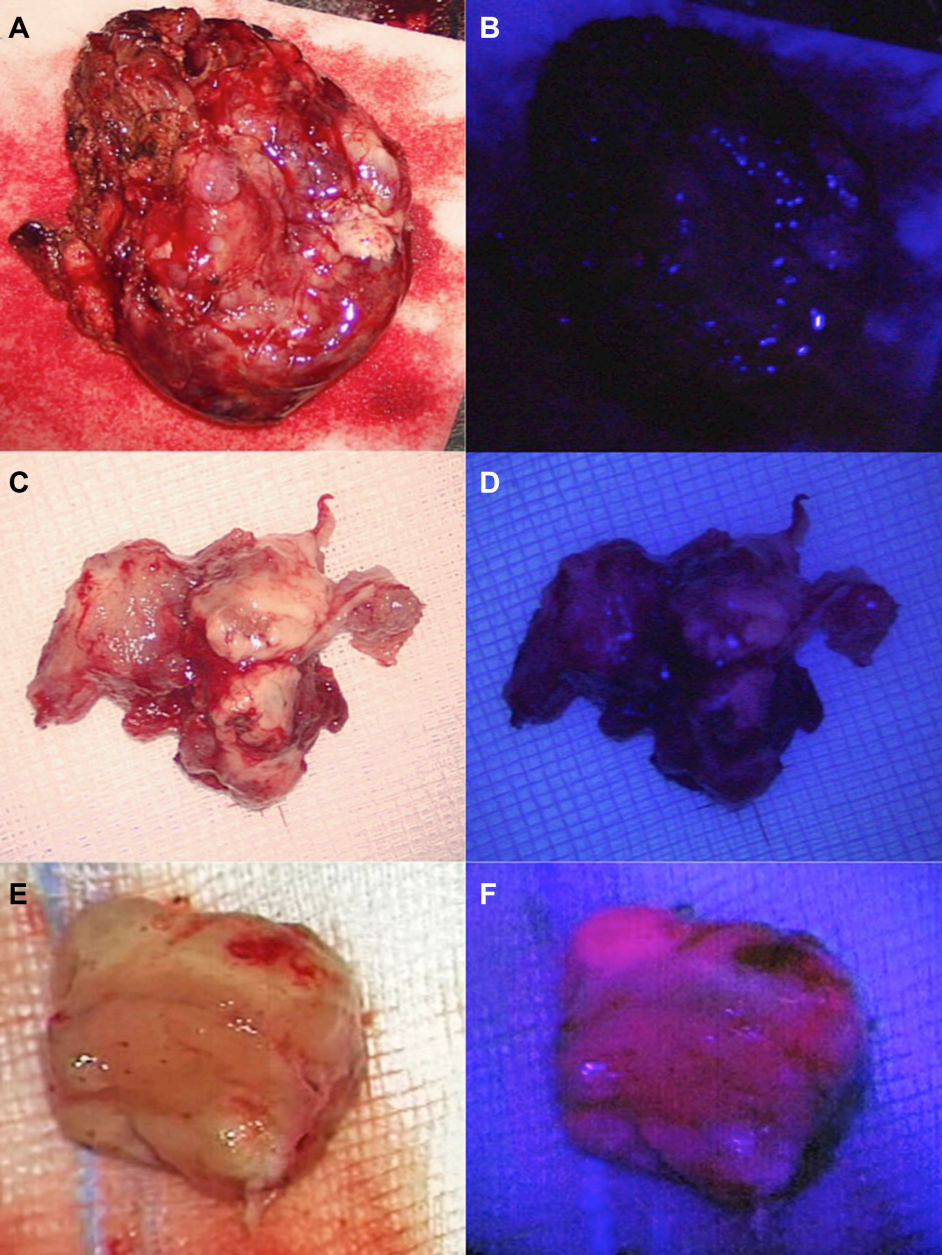
Different shades of 5-ALA-induced fluorescence of cerebral metastases Cerebral metastases may appear as ALA- negative (**A**, **B**) or faintly (**C**, **D**) or strongly ALA-positive (**E**, **F**).

**Figure 2:**
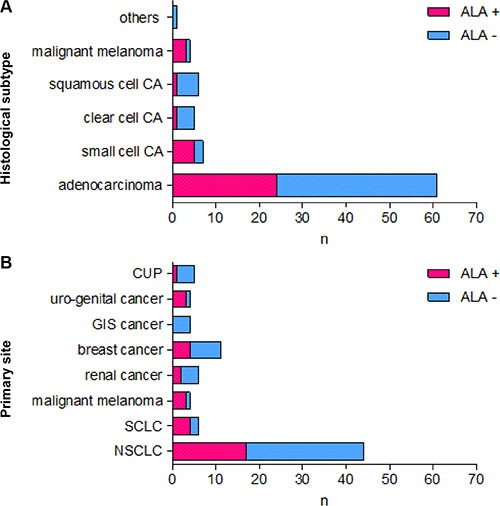
5-ALA-derived fluorescence behavior of cerebral metastases according to their histological subtype and the primary site Figure 2 shows the 5-ALA-derived fluorescence behavior of cerebral metastases according to their histological subtype (**A**) and the primary site (**B**).

34 cerebral metastases (40.5%) showed either strong or faint and 50 metastases (59.5%) no 5-ALA derived fluorescence (Figure 1). Neither the primary site of the cerebral metastases (*p* = .24, Fisher's Exact Test) nor its histology (*p* = .17, Fisher's Exact Test) statistically correlated with the fluorescence behavior (Figure 2).

Extend of surgical resection was evaluated by an early postoperative MRI within 72 hours after surgery: A complete surgical resection was achieved in 47 patients (56%) and residual tumor was detected in 15 patients (17.9%). In 22 metastases (26.1%) the degree of resection could not accurately be determined. Dichotomized 5-ALA fluorescence (yes vs. no) had no statistical influence on the degree of surgical resection (*p* = .52, Fisher's Exact Test).

### 5-ALA derived fluorescence, local in-brain-progression and overall survival

General in-brain-progression was observed in 33/84 patients (39.3%). 27 patients (32.1%) developed an in- brain-progression at distant sites and 8 patients (9.5%) a leptomeningeal carcinomatosis.

**Figure 3:**
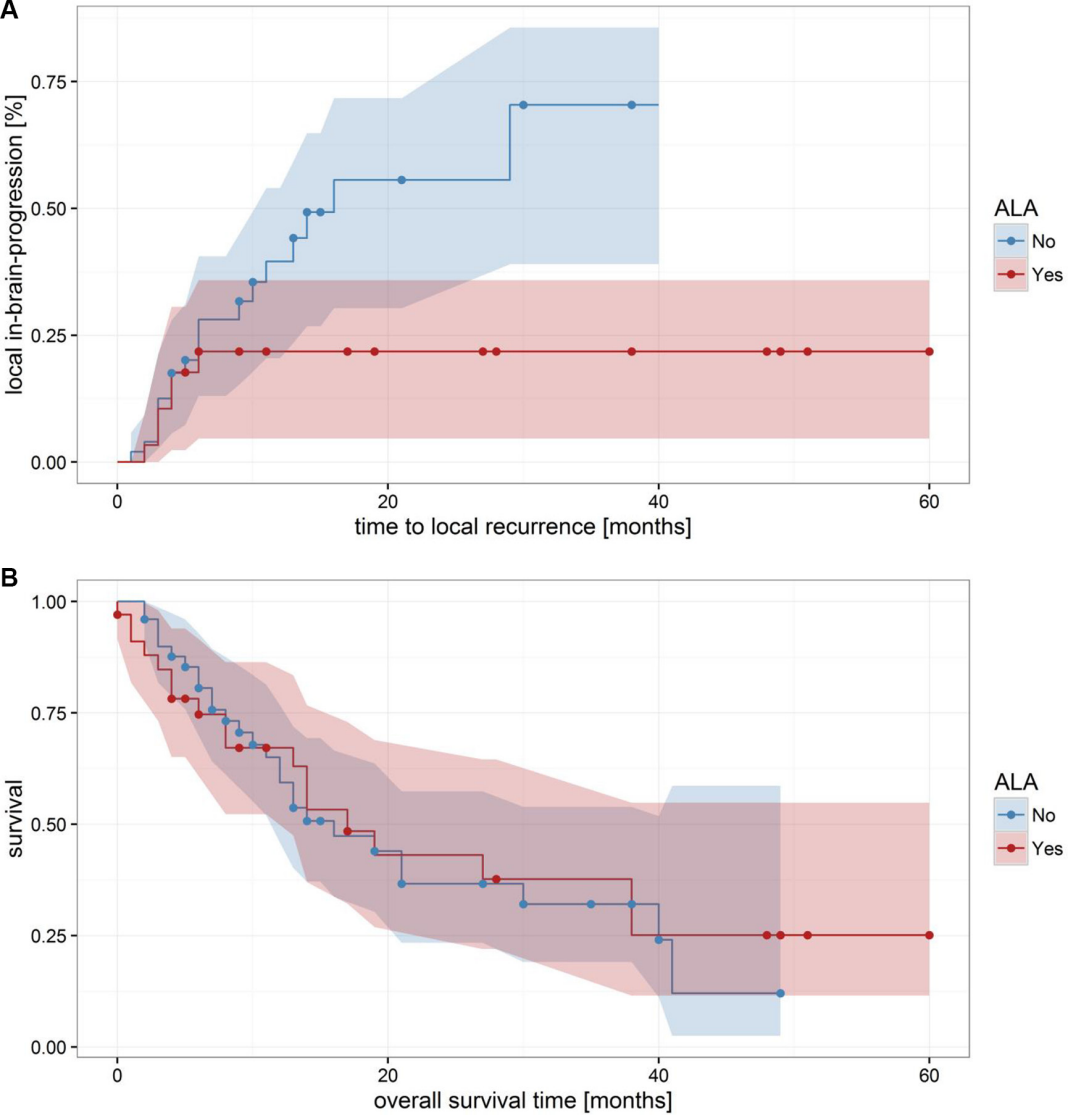
Local in-brain progression and overall survival (**A**) shows significant differences in the local in-brain-progression rate of 5-ALA positive and 5-ALA negative metastases (*p* = 0.0456). In contrast, the 5-ALA fluorescence behavior had no significant influences on the overall survival (**B**) *p* = 0.852).

Local in-brain progression within or at the border of the resection cavity was observed in 26 patients (30.9%). Of these 26 metastases with a local tumor recurrence, 6 (23.1%) showed 5-ALA derived fluorescence and 20 (76.9%) were ALA-negative. In our present series, 5-ALA derived fluorescence of metastases had a significant influence on the local in-brain-progression rate (*p* = 0.03; Fisher's Exact Test; 95-CI: 0.093–0.99; odds ratio: 0.32). Mean time to local progression was 7.6 ± 1.5 m (1–29 m); 3.6 ± 0.6 m for ALA-positive metastases and 8.9 ± 1.8 m for ALA-negative metastases. The time to local in-brain-progression was log-normally distributed (Shapiro-Wilk normality test: *p* = 0.84; *p* = 0.8 for ALA-positive and ALA-negative, respectively) and differed significantly between both groups (*p* = 0.03; Welch Two Sample *t*-test; 95-CI: 0.063–1.097; Figure 3). The curves depicting the progression also differed significantly (Mantel-Haenszel log-rank test: *p* = 0.046).

The mean overall survival was 14.7 ± 1.5 m (0– 60 m), no significant difference was found with respect to the fluorescence behavior (*p* = 1; Fisher's Exact Test).

### Local in-brain-progression and overall survival after radiation therapy

Adjuvant radiation therapy is known to significantly influence the rate of local in-brain progression. Therefore, a possible relation of the 5-ALA derived fluorescence behavior of cerebral metastases and the local in-brain progression rate, the time to tumor recurrence and the overall survival was re-evaluated for the subgroup of patients scheduled for an adjuvant radiation therapy (*n* = 64).

**Figure 4:**
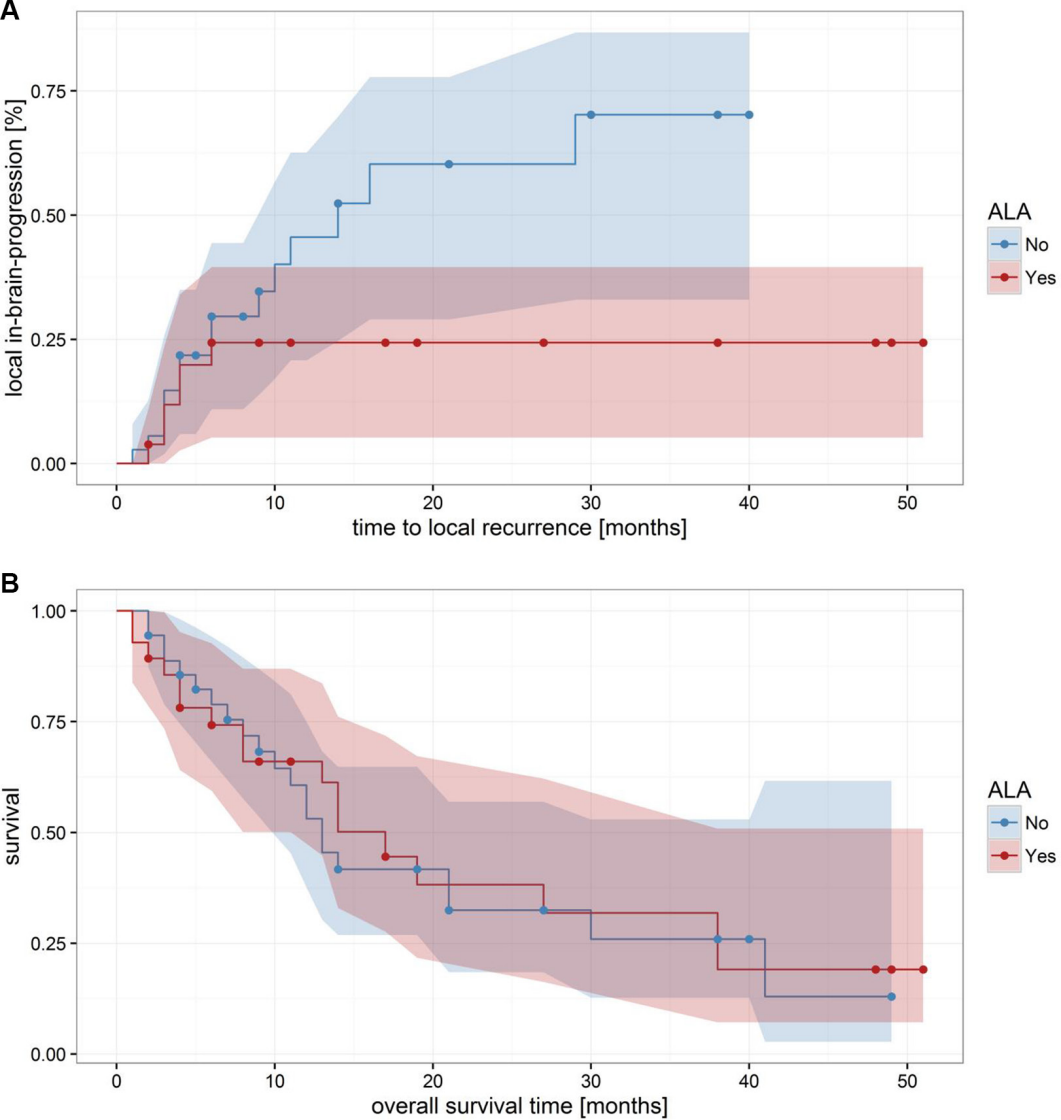
Local in-brain-progression and overall survival after radiation therapy After exclusion of the 20 patients without any form of adjuvant radiation therapy, there was a trend towards an effect of the 5-ALA behavior of cerebral metastases on the local recurrence rate and the time to local recurrence, although results did not anymore reach significance.

After exclusion of the 20 patients without any form of adjuvant radiation therapy, there was a trend towards an relation of the 5-ALA behavior of cerebral metastases on the local recurrence rate and the time to local recurrence, although results did not reach the postulated level of significance anymore (for the local-in-brain-progression rate: *p* = 0.1; Fisher's Exact Test; 95-CI: 0.1–1.3; for the time to local in-brain progression: *p* = 0.089; Welch Two Sample *t*-test; 95-CI: -0.08–1.1). No significant difference between the fluorescence groups regarding mean overall survival time (*p* = 0.74, Welch Two Sample *t*-test) or on the survival curves was found (*p* = 0.86, Mantel-Haenszel log-rank test; Figure 4).

## DISCUSSION

The main findings of this retrospective analysis of the 5-ALA fluorescence in cerebral metastases are that (1) approximately 40% showed a faint or strong 5-ALA-induced fluorescence, (2) the dichotomized 5-ALA behavior was neither related to the extend of surgical resection nor to the overall survival and (3) a higher local-in-brain progression rate for 5-ALA-negative metastases as compared to 5-ALA-positive metastases is seen. After exclusion of those patients without an adjuvant radiation therapy, a weak relation between fluorescence behavior, local recurrence rates and the time to local recurrence was seen, although results did not reach statistical significance.

Application of 5-ALA allows an intraoperative detection of malignant glioma tissue with high sensitivity and specificity. 5-ALA fluorescence guided resection leads to a more radical tumor resection as compared to conventional techniques which leads to an increased progression free survival [16–18]. 5-ALA induced fluorescence of neoplasms has been observed for various malignancies outside the central nervous system, such as skin, bladder, and colorectal cancer. Several diagnostic and therapeutic 5-ALA-based concepts have been discussed [28–35]. Therefore, investigation of its use in the resection of cerebral metastases seems justified and worthwhile.

However, only 40% of cerebral metastases in the present series exhibited either faint or strong 5-ALA induced fluorescence, which is in good agreement with previous studies: Utsuki and coworker were the first to analyze 5-ALA induced fluorescence of cerebral metastases; in their series 9/11 metastases (82%) were ALA-positive [19]. Since then, two series (each including more more than 50 patients) found positive 5-ALA fluorescence in 52% and 62% of cerebral metastases [15, 20].

5-ALA fluorescence guided resection of cerebral metastases was evaluated as a tool to detect residual metastatic tumor tissue after macroscopically complete resection. Such unintended remnants of metastatic tumor tissue can frequently be detected by early postoperative MRI and might be one reason for the high local recurrence rate after surgical resection [13]. However, the sensitivity of 5-ALA fluorescence-guided resection for the identification of such residual tumor is limited: In a previous study, 57.2% of patients showed residual 5-ALA fluorescence after gross-total resection of a cerebral metastasis whereas residual tumor cells in biopsies from these areas were confirmed in only 1/3 of patients [15].

Interestingly and unexpectedly, we observed a significant correlation between the dichotomized 5-ALA fluorescence behavior of cerebral metastases and its local recurrence rate. This correlation did, however, not reach significance any longer after exclusion of patients without any form of radiation therapy–although a trend remained. This trend might reach significance in greater patient collective. Further studies or a meta-analysis of survival data of the yet published studies might address this issue.

One possible explanation for a relation between the 5-ALA signal and local recurrence rate is that 5-ALA positive metastases were more radically resected than 5-ALA negative metastases. The surrounding tumor-free tissue of cerebral metastases partially show 5-ALA derived fluorescence which might lead to unintended more radical resections [15, 19, 36] and the degree of surgical resection is known to correlate with the local recurrence rate [21, 37]. 5-ALA fluorescence of the macroscopically tumor-free adjacent brain tissue was also observed in some studies. 5-ALA fluorescence of the macroscopically normal appearing resection cavity was observed in single patients included in the present study. However, we neither documented systematically the 5-ALA fluorescence of the resection cavity nor resected this fluorescent tissue for a further histopathological evaluation as this tissue might be functional [8, 15, 21, 38]. In our present patient population, the degree of surgical resection was not significantly different as assessed by an early postoperative MRI between 5-ALA-positive and -negative metastases. A higher rate of unintended “supramarginal resections” in the group of 5-ALA-positive metastases cannot be excluded as resections beyond the margins of the contrast-enhancing metastasis can, until now, not sufficiently be quantified in postoperative MRIs [21].

**Figure 5:**
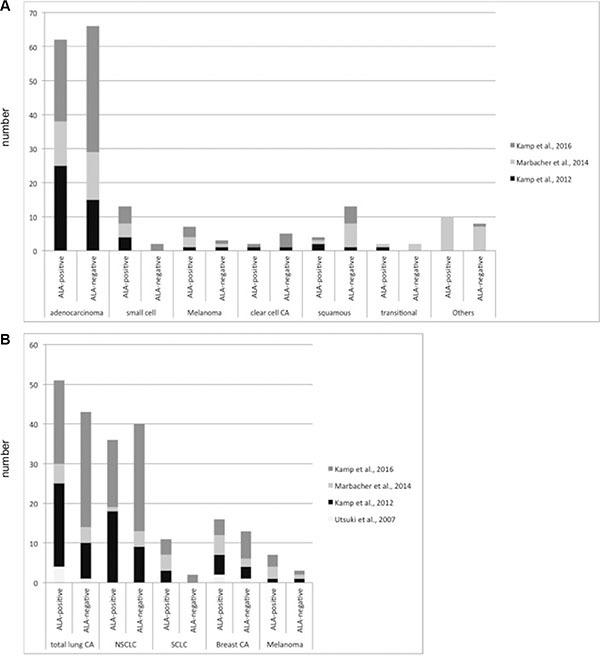
5-ALA-derived fluorescence behavior of cerebral metastases according to their histological subtype and the primary site in the three recent studies with more than 50 patients

Another explanation for the higher local recurrence rate of 5-ALA-negative metastases might be a yet not identified intrinsic factor reflecting a more aggressive behavior of these metastases: 5-ALA-derived fluorescence of cerebral metastases neither correlated with their primary site nor its histological subtype in two previous studies and the current series (Table 2; Figure 5) [15, 20]. However, heterogeneity of cerebral metastases with the same histology and origin is well known [39–41]. Several molecular markers, e.g. such as BRAF expression in malignant melanoma, are well known to correlate with aggressiveness of the tumor and its prognosis. The 5-ALA fluorescence behavior might therefore be a similar marker, which correlates with aggressiveness of the tumor: 5-ALA is a natural precursor molecule of the mitochondrial heme synthesis pathway and is converted into protoporphyrin IX (PpIX). PpIX is the actual fluorescent agent capable of fluorescence after blue light excitation. 5-ALA-induced fluorescence of malignant glioma and some cerebral metastases is probably caused by an abnormal accumulation of PpIX in the mitochondria. In normal tissues, iron ions are incorporated into PpIX by the ferrochelatase. A lower ferrochelatase activity in malignant tumor tissue was proposed to be responsible for an accumulation of PpIX. In fact, ferrochelatase downregulation and loss of enzymatic activity correlates with an enhanced PpIX-dependent fluorescence in gastric and colorectal cancer cell lines [42]. A heterogeneous downregulation of the ferrochelatase in different patients and even within an individual metastasis might be responsible for a heterogeneous 5-ALA-induced fluorescence behavior of cerebral metastases. One possible explanation for a heterogeneous ferrochelatase expression might be a heterogeneous oxygenation within cerebral metastases. Such a heterogeneous pattern of oxygenation is a key feature of many solid malignant tumors [43–45]. Hypoxia has a direct influence on the ferrochelatase expression by a mechanism involving the hypoxia-inducible factor 1 (HIF-1) [46]. Furthermore, a recent gene expression profiling linked the outcome of patients with desmoid tumors with the ferrochelatase expression. Overexpression of ferrochelatase was found in more aggressive forms of desmoid tumors and was associated with a poor clinical outcome [47].

**Table 2:** Summary of studies analyzed 5-ALA fluorescence behavior of cerebral metastases

First author	Journal	Year	patients with cerebral metastases	5-ALA positive	%
Utsuki	Brain Tumor Pathol	2007	11	9	81.8 %
Hefti	SwissMedWkly	2008	2	2	100%
Valdés	JNS	2011	3	6/12 samples	50%
Schucht	Acta Neurochirurg	2011	1	0	0%
Kamp	Acta Neurochirurg	2012	52	32	61.5 %
Widhalm	Neurosurg Rev	2012	4 pts. (6 samples)	3/6 samples (vague)	50%
Suero Molina	Clin Neurol Neurosurg	2013	7	7	100%
Eljamel	Photodiagnosis Photodyn Ther.	2013	5	5 (mixed, not red intense)	100%
Marbacher	Neurosurg Focus	2014	65	34	52%
Belloch	Acta Neurochirurg	2014	3	1	33.3%
Piquer	BioMed Research International	2014	3	3	100%
Coburger	Neurosurg Focus	2014	11	8	72.7%

We have to acknowledge several limitations of our present study: (1) the results of our analysis are limited by the retrospective study design and results arise from a single center study. (2) As many retrospective studies analyzing cerebral metastasis, our present study includes a heterogeneous patient population with patients suffering from different primary tumors, different tumor stages and different adjuvant therapy concepts. Patients were treated by different types of adjuvant radiation therapy after surgical resection, which might have an influence on the local in-brain progression rate. One reason for the different radiation concepts is–again–the heterogeneity of different primary tumors, degree of surgical resection, number of metastases and postoperative neurological performance. Furthermore, the EORTC 22952–26001 study was published in 2011 and the additional analysis of quality of life data in 2013, which is within the observation period of the present study [9, 48]. The EORTC 22952– 26001 study showed a significantly reduced incidence of local in-brain-progressions after WBRT as compared to observation but also a significant negative impact on the patient's quality of life and cognitive function after WBRT without an improved overall survival [9, 48]. Therefore, the EORTC 22952–26001 trail led to a change in the recommendations for an adjuvant radiation therapy after metastases resection. As adjuvant radiation therapy is known to significantly influence the rate of local in- brain progression, we analyzed the impact of the 5-ALA derived fluorescence behavior of cerebral metastases on the local in-brain progression rate for the subgroup of patients scheduled for an adjuvant radiation therapy. A differential effect of different adjuvant radiation protocols (e.g. WBRT vs. local fractionated cavity boost radiation) is not known. However, in view of the great heterogeneity of the study population and applied treatment modalities the number of patients to be analyzed in further subgroups (e.g. WBRT vs. cavity boost radiation vs. stereotactic radiosurgery vs. observation) is rather too small for a valid statistical analysis. (3) Besides the parameters analyzed here, many other factors might additionally influence the local-brain progression rate. Such factors include but are not limited to the preoperative tumor volume, the growth pattern of cerebral metastases or the mode of surgical resection (piecemeal vs. *en bloc* resection) [49–51]. The degree of resection could not accurately be determined by a postoperative MRI with 72 hours in 26.1% of cerebral metastases in the present studies. A reliable estimation of the resection degree might be difficult for several reasons: Residual tumor tissue could not be reliably differentiated from dilated vessels in the wall of the resection cavity, residual melanin of melanoma metastases could hardly be distinguished from postoperative blood or image quality might be impaired e.g. by patient motion). In a previous study, no final decision on degree of resection could be made in nearly 20% of metastases (18.5 %) [13]. Reducing this uncertainty represents a challenge to neuroradiology. (5) In the present study, all patients with newly diagnosed unclear cerebral lesions were subjected to preoperative administration of 5-ALA if intraoperative diagnosis of a malignant glioma was possible. The technique of 5-ALA-guided resection of malignant gliomas is standard in many neuro-oncological departments and results in more complete surgical resections and subsequently in a prolonged progression-free survival as compared to standard microsurgical resections. A reliable pre-operative differentiation between malignant glioma and cerebral metastasis is not yet possible. Even in patients with known malignant disease a glioma should always be considered as a differential diagnosis [52]. As patients with a misdiagnosed malignant glioma will lose the benefits of the 5-ALA technique and subsequently have a significantly worse prognosis, we and others use 5-ALA for any lesion that might be a malignant glioma [52, 53] (6) 5-ALA fluorescence was values by the surgeon and was dichotomized into fluorescent or non-fluorescent. Such a dichotomized categorization of 5-ALA fluorescence is simple and common as it is used in over 60% of studies reporting on the impact of 5-ALA-derived fluorescence [54]. However, two different fluorescence types of 5-AIF were initially defined [55]: a deep red fluorescence designated as “solid” and less intense “vague” fluorescence. A quantitative estimation of 5-ALA fluorescence might give more objective value. However, this is beyond the scope of the present work and the 5-ALA technique as it was initially applied.

Less than half of cerebral metastases show 5-ALA-induced fluorescence in the present series and the 5-ALA fluorescence behavior observed was neither linked to histological pattern nor to a certain primary site. The dichotomized 5-ALA fluorescence had no statistical influence on the degree of surgical resection. Absence of 5-ALA-induced fluorescence was a risk factor for local in-brain-progression but did not influence the mean overall survival. The dichotomized 5-ALA fluorescence behavior might be an indicator for a more aggressive biological behavior in cerebral metastases.

## MATERIALS AND METHODS

### A retrospective evaluation was performed to evaluate 5-ALA-induced fluorescence of cerebral metastases

The charts of all patients who underwent resection of a cerebral metastasis between 11/2010 and 12/2015 at our tertiary care center were reviewed. All patients included in the present analysis fulfilled the following criteria: (1) surgical resection of a cerebral metastasis, (2) intraoperative estimation of 5-ALA-induced fluorescence by the neurosurgeon, (3) histopathological confirmation of a cerebral carcinoma or malignant melanoma metastasis and (4) first diagnosis of a cerebral metastatic spread. Exclusion criteria were other tumors than cerebral carcinoma or melanoma metastasis (e.g. patients suffering from glioma, cerebral lymphoma or e.g. sarcoma metastases), biopsies or standard white-light assisted surgical resection without an intraoperative estimation of the 5-ALA-induced fluorescence as well as surgery of recurrent metastases.

### 5-ALA

5-aminolevulinic acid (5-ALA) was obtained by the hospital pharmacy from Caelo (Caesar & Loretz GmbH, Hilden, Germany).

### Surgery

All patients with newly diagnosed unclear cerebral lesions were subjected to preoperative administration of 5-ALA if diagnosis of a malignant glioma was possible. 5-ALA was administered for all patients 3 h prior to surgery in a dose of 20 mg per kilogram body weight as described before [15, 17]. Intraoperative frozen sections were obtained in all patients. After the histological diagnosis of an intracerebral metastasis by frozen section, standard white-light assisted–and if possible–*en bloc* circumferential resection was performed [15]. 5-ALA fluorescence was visualized by the surgical microscope equipped with a 5-ALA fluorescence detection tool (OPMI Pentero microscope with the FLOW 800 tool; Carl Zeiss Meditec, Oberkochen, Germany or the Leica M530 OH6 microscope, Leica Microsystems GmbH, Wetzlar, Germany). 5-ALA fluorescence was valued by the surgeons and dichotomized classified in 5-ALA fluorescent (any kind of 5-ALA fluorescence, either weak or strong) or 5-ALA non-fluorescent. In the case of intraoperative diagnosis of a brain metastasis after analysis of the frozen section and positive 5-ALA fluorescence, we documented the fluorescence status of the metastases and performed a standard white-light assisted resection. For patients with eloquently located metastasis, surgery was performed with intraoperative monitoring and as awake surgery in an asleep-awake-asleep protocol [21]. Eloquent brain regions were defined as a cortical or subcortical brain area at which we expect intraoperative stimulation to elicit changes in neurologic condition (particularly regarding speech, movement and tactile sensation) or to elicit a response in electrophysiological recordings in corresponding areas [21].

### Pre-/postoperative imaging, data collection, follow-up and definition of outcome measures

Preoperative imaging was performed by an contrast-enhanced 1,5T-MRI (Avanto, Siemens, Erlangen, Germany) [13]. Extent of surgical resection was verified by an early postoperative contrast-enhanced 1,5T-MRI within 72 hours after surgery using the same sequences. Residual contrast-enhancing parts in the T1 sequences as well as T2 and diffusion sequences were examined for residual tumor [13]. Standard evaluation blood examination including liver enzymes was performed before surgery and on the third postoperative day.

Using an integrated medical-record system, we performed a retrospective analysis of data on patients fulfilling the inclusion criteria. Epidemiological data (age, gender), data regarding tumor location, the primary site, surgical technique and pre- and postoperative images were collected. Recommendations for a further adjuvant treatment after metastasis resection were usually discussed in an interdisciplinary tumor board. Recommendations for the type of adjuvant radiation depended on various parameters as number of cerebral lesions, degree of resection, KPS and will of the patients. Types of adjuvant radiation therapy are summarized in Table 1. Follow-up was scheduled every 3 month after surgery including a contrast-enhanced MRI. Mean follow-up time was 14.7 ± 1.5 month.

Local in-brain-progression was defined as a tumor recurrence or progression within or at the border of the resection cavity according to the RANO criteria [22]. Distant in-brain-progression was defined as occurrence of new contrast-enhancing lesions in the postoperative or follow-up MRIs distant to the site of the resected metastasis. Leptomeningeal carcinomatosis was either diagnosed by detection of malignant tumor cells in the cerebro-spinal fluid or by a diffuse nodular tumor progression of meninges on MRI. Time to (local) in- brain-progression is defined as time between surgery and diagnosis of the (local) in-brain-tumor progression. Overall survival was defined as the timespan from surgery to death.

### Statistical analysis

For categorical data—ALA fluorescence, tumor kind, residual tumor etc.—the Fisher's exact test was used to test the independence of variables [23]. The test computes the probability of obtaining the observed contingency table or a table with more pronounced dependencies, but having the same row and column sums, under the null hypothesis that the variables are independent.

Real-valued data—time to local or distal recurrence, survival time etc.—were first tested for normality using the Shapiro-Wilk normality test [24]. Its null hypothesis is that the population from which the sample is taken is normally distributed. If the variables were found to have no significant deviation from the normal distribution, either on the linear or on the logarithmic scale, the Welch's *t*-test was used to compare the variable distributions [25]. The Welch's *t*-test is a variant of the Student's *t*-test for populations with unequal variances [26]. If the variables themselves were normally distributed, the *t*-test was applied directly to the data to check whether the distribution means significantly differ. For variables, which more closely fitted the log-normal distribution, the *t*-test was applied to the logarithmically transformed variables. No real-valued variable was found to significantly deviate from normality both on the linear and on the logarithmic scale.

The Kaplan-Meier survival curves were compared using the log-rank test, also known as the Mantel-Haenszel or Mantel-Cox test [27]. The test statistics relies on the differences between the observed and expected number of events (i.e. deaths) at all times under the null hypothesis that there is no difference between the two groups. The test treats censored data in the same manner as the Kaplan-Meier curves and is therefore particularly suitable for comparing them. The *p*-value is the probability of obtaining the same or more extreme value of the test statistics from the corresponding χ^2^ distribution.

This retrospective, uncontrolled single center analysis was approved by the local Research Ethics Committee and institutional review board (internal study number: 3307 and 5269).
